# Digital health promotion, prevention and health literacy measures among seafarers and crews on cargo and passenger ships: A scoping review

**DOI:** 10.1371/journal.pone.0356798

**Published:** 2026-08-26

**Authors:** Chiara Reck, Lukas Belz, Dorothee Dengler, Nora Maria Puls, Volker Harth, Marcus Oldenburg

**Affiliations:** Department of Maritime Medicine, Institute for Occupational and Maritime Medicine (ZfAM), University Medical Center Hamburg-Eppendorf, Hamburg, Germany; Tarbiat Modares University Faculty of Medical Sciences, IRAN, ISLAMIC REPUBLIC OF

## Abstract

**Introduction:**

The unique occupational conditions of seafaring are associated with increased physical and psychological stress and significantly complicate common approaches to health promotion and prevention. Digital health technologies are therefore considered promising alternatives that can better address barriers to access, infrastructural shortcomings and cultural diversity.

**Objective:**

The aim is to provide a comprehensive overview of scientific evidence about digital measures used for health promotion and prevention among seafarers and crew members on merchant ships (cargo/passenger). As secondary study aim, digital practical programs established to promote health at sea will also be presented.

**Methods:**

This scoping review was conducted in accordance with the updated methodological guidelines from the Joanna Briggs Institute (JBI) and the PRISMA-ScR checklist. The protocol was prospectively registered in the Open Science Framework (OSF). The literature search was conducted systematically in Scopus, Web of Science, PubMed, and LIVIVO, and study selection was carried out based on the selection criteria. In addition, a targeted web search for digital practical programs was conducted.

**Results:**

A total of 1,711 sources were identified, 68 full-text articles were screened, and 9 studies were included in the qualitative analysis. The evidence base is highly heterogeneous, consisting of various study designs ranging from pilot studies to one randomized controlled trial (RCT). Only articles on digital health promotion and prevention were identified. No studies were found that examined digital health literacy. All included studies focused exclusively on seafarers working on cargo ships. Four studies investigated behavioral approaches, while two studies focused on structural approaches and the remaining three studies dealt with blended concepts. Thematic focus varies, addressing topics such as nutrition, mental health, skin cancer prevention, and digital prediction models. In addition, six digital practical programs on topics such as physical activity, mental health, sleep and stress management, and nutrition were identified.

**Conclusions:**

An overview of the current state of research and practical applications regarding digital health promotion and prevention among seafarers on merchant ships was provided. The topic has been scarcely addressed in the shipping industry to date. Concurrently, the heterogeneity of the evidence, the restricted number of relevant studies, and the variable quality of the studies indicate a necessity for further research. Academic research should also address the topic of digital health literacy and the demands of crew members on passenger ships. Based on the findings, it is recommended that shipping companies take responsibility for promoting the health of seafarers on board their ships by empowering their crews.

## 1. Introduction

The maritime industry is a cornerstone of global trade, employing millions of seafarers and crew members on cargo and passenger ships worldwide. They work and live in environments that are fundamentally different from land-based conditions, characterized by prolonged periods at sea, isolation from family and friends, multicultural crew compositions, confined spaces and limited access to health facilities [1,2]. With the introduction of the International Ship and Port Facility Security Code (ISPS-Code) in 2004, the conditions for shore leave and therefore the possibility to use land-based health facilities for seafarers have deteriorated significantly. This is because the code has led to a more restrictive approach to shore leave conditions for seafarers, even though the code formally mandates that shore leave must be facilitated [3]. Furthermore, the pace of shipping, with shorter port stays and a significant increase in workload, is another critical factor that reduces the chance for shore leave [4]. The unique occupational context of seafaring presents significant challenges for the implementation of conventional health promotion and prevention strategies and has been associated with increased risks for both physical and mental health problems [5,6].

Despite the well-documented health risks faced by seafarers, including fatigue, stress, musculoskeletal disorders, and mental health issues, traditional health interventions are often impractical or ineffective in the maritime setting due to logistical constraints and limited resources [7,8]. In response, offerings from the field eHealth, meaning the use of information and communication technologies (ICT) to support health, and mHealth, meaning the use of mobile wireless technologies for health, provide remote opportunities to reach the target group of multicultural seafarers at sea [9]. Thus, digital health technologies have emerged as promising alternatives, offering the potential to overcome barriers related to distance, infrastructure, and cultural diversity [7]. Digital health is used as an umbrella term and is broadly defined as “the field of knowledge and practice associated with the development and use of digital technologies to improve health”, and encompasses a range of digital technologies, including eHealth, telemedicine but also artificial intelligence (AI), ‘big data’ and robotics [10].

Health promotion, as defined by the World Health Organization (WHO) in 1986, refers to strategies and interventions aimed at enabling individuals and communities to increase control over and improve their health [11]. Prevention is characterized as the implementation of measures aimed to both avert the occurrence of disease and risk factor reduction or mitigate its impact [12], while health literacy is defined as the ability to access, understand, appraise, and apply health information to make informed health-related decisions [13]. In the maritime context, these concepts can only be operationalized through interventions that are accessible remotely, adaptable to diverse cultural backgrounds, and feasible within the constraints of shipboard life. In comparison with shore-based cohorts, maritime workers encounter a unique set of health challenges.

In recent years, there seems to be a growing interest in digital health solutions for seafarers, with initiatives ranging from telemedical support and online mental health resources to interactive training modules and health information platforms [14]. For example, ISWAN provides online health materials [15] or Sailor’s Society developed the mobile app “My Wellness at Sea” [16]. However, the available evidence on digital health promotion, prevention, and health literacy for seafarers appears to be both limited and highly heterogeneous. While there are articles addressing seafarers’ health promotion in general by conducting surveys for needs analyses [14,17], other studies addressed specific health issues, such as skin cancer preventive behavior [18] or mental health issues [19]. Some study designs appear to be limited to only seafarers from specific regions or working on vessels flagged by a certain nation or belonging to a shipping company [14,18,20].

Given these limitations, this article adopts an inclusive approach, considering not only scientific evidence and grey literature within the scoping review, but also providing insights into the landscape of digital health programs that have been implemented in practice without formal scientific evaluation. By mapping both the scientific and practical aspects, this review aims to provide a comprehensive overview of digital health offerings for seafarers and crew members in merchant shipping, whilst also identifying existing gaps in this crucial yet under-researched domain. To the current authors’ knowledge, the extant literature includes no literature reviews dealing with the scope of the present article.

The following research questions were formulated with the intention of guiding this scoping review.

### Primary research question

- What digital applications and measures for health promotion, prevention, and health literacy development are described and implemented for seafarers and crew members on cargo and passenger ships?

### Secondary research questions

- How are the applications and measures accepted and enjoyed by the target group?
- What practical programs exist for seafarers and crew members on civilian cargo and passenger ships?

## 2. Methods

This review was conducted as a scoping review, following the updated methodological guidance from the Joanna Briggs Institute (JBI) [21] and reported in accordance with the PRISMA Extension for Scoping Reviews (PRISMA-ScR) checklist (S4 File) [22]. The scoping review approach was selected to map the existing literature regardless of study design or methodological quality.

The protocol for this review was prospectively registered on the Open Science Framework (OSF) [23], ensuring transparency and reproducibility. All methodological decisions, including eligibility criteria, search strategy, and data extraction procedures, were documented in the registration.

### 2.1 Eligibility criteria

The inclusion and exclusion criteria for this scoping review were established using the PCC (Population, Concept, Context) framework as recommended by Peters et al. (2020) [21]. Studies were eligible for inclusion if they focused on seafarers and crew members working on cargo or passenger ships, and described digital health promotion and prevention, or digital health literacy, to provide an overview of digital health in the maritime context as comprehensive as possible. Literature published in English or German between January 2015 and August 2025 with full-text availability were considered. The 10-year time span was determined in consideration of the rapid rate of change in the field of digital health technologies. In order to provide a comprehensive overview of the full range of developments, no type of literature was excluded. Furthermore, grey literature is also encompassed, for example, bachelor’s and master’s theses, project reports, and conference papers. Moreover, the review includes websites and practical program reports of available digital health offers for seafarers and crews, with the aim of mapping current actions in this field.

Exclusion criteria were applied to studies focusing on military or naval sectors, offshore workers, port workers, and fishers. Additionally, sources focusing on therapy, clinical treatments, or telemedicine for acute care, as well as those addressing morbidity, mortality, or injuries on board were excluded. Literature limited to safety or ship technology topics, or without full-text access, was also not considered for review.

### 2.2 Search strategy

A full search strategy for the review was developed, including the decision for the eligible databases and creation of the final search strings for each database (S1 File). A comprehensive search was conducted between August 14, 2025, and September 02, 2025, in the following electronic databases: PubMed/MEDLINE, Scopus, Web of Science, and LIVIVO (ZB MED Search Portal for Life Sciences), in which searches in PSYNDEX, BASE, ZB Sport, bibnet.org, and relevant catalogues (NLM, TIB, ZB MED) were included.

For example, the Scopus query was:

TITLE-ABS-KEY((seafarer* OR sailor* OR “ship crew*” OR “ship personnel*” OR seaman OR seamen) AND (ship* OR “merchant vessel*” OR ferry OR ferries OR “cruise ship*” OR “at sea*” OR onboard OR “on board” OR “merchant marine*” OR seagoing) AND (“health literacy” OR “health competence” OR “health education” OR “health promotion” OR prevention OR “health measure*” OR “health intervention*” OR “health program” OR ehealth OR “electronic health” OR mhealth OR “mobile health” OR dhealth OR “digital health”))

The reference lists of all included resources were screened for additional studies. Further literature was identified through hand searches of the institutional internal maritime medical documentation, WHO and IMO reports, conference proceedings (e.g., International Maritime Health Symposium) and Google Scholar searches. Backward citation searching of included studies was also performed.

### 2.3 Study selection

All identified records were imported into EndNoteTM 20 (Clarivate) and deduplicated. Screening was conducted in two stages using Rayyan [24]: (1) title and abstract screening, and (2) full-text screening. A single reviewer performed all screening steps due to limited personnel resources, consulting co-authors in cases of uncertainty. Exclusion decisions were documented with justification, and the screening process was reported using a PRISMA-ScR flow diagram.

### 2.4 Data extraction, analysis and presentation

Data extraction for this review was conducted using a predefined extraction matrix in Microsoft Excel 365.

From each included source, the following data items were systematically charted: publication metadata (including study type, title, authors, year, country, journal, and impact factor); primary target parameter; population characteristics (description, inclusion criteria and exclusion criteria), concept-related (intervention, media and technology used, outcome variables, results), context, and methodological details, and presented in tables as well as accompanied by narrative summarization related to the reviews objective and research questions.

### 2.5 Methods of quality appraisal

Methodological quality appraisal of included studies was conducted using the Mixed Methods Appraisal Tool (MMAT) version 2018, selected for its applicability to diverse study designs including qualitative, quantitative, and mixed methods research. This tool enables a streamlined critical appraisal across study types relevant to scoping reviews, where heterogeneous evidence is common, without generating overall quality scores as per MMAT recommendations [25]. To represent the overall quality of the references, a percentage score from 0 to 100% is calculated according to the instructions provided by the MMAT authors.

The AACODS Checklist (Authority, Accuracy, Coverage, Objectivity, Date, Significance) was used for grey literature and reviews. It was developed to appraise non-scientific sources, ensuring a comprehensive evaluation aligned with scoping review principles [26]. To maintain clarity in the quality assessment, reviews that cannot be assessed using the MMAT, are instead evaluated also using the AACODS checklist. This dual-tool approach enables quality assessment of all included resources.

No studies were excluded based on quality assessment. The objective of employing these appraisal tools is to present the quality of the included studies and to provide a more comprehensive understanding of the body of existing literature in this field.

### 2.6 Web search for digital practical health programs

To supplement the scientific findings, an online search was conducted to identify digital health promotion programs for seafarers that are already being implemented in practice. The web search was carried out by hand in two search engines (Google and Ecosia) after the systematic evidence search and data extraction, on December 10, 2025. The same keywords were used as in the query string listed above.

## 3. Results

This section presents the outcomes of the systematic literature search, hand search and study selection process, conducted in accordance with PRISMA guidelines to ensure transparency and reproducibility, as well as an overview of digital health programs that can be found online specifically for seafarers and crew members of merchant vessels.

### 3.1 Study characteristics

**Fig 1:**
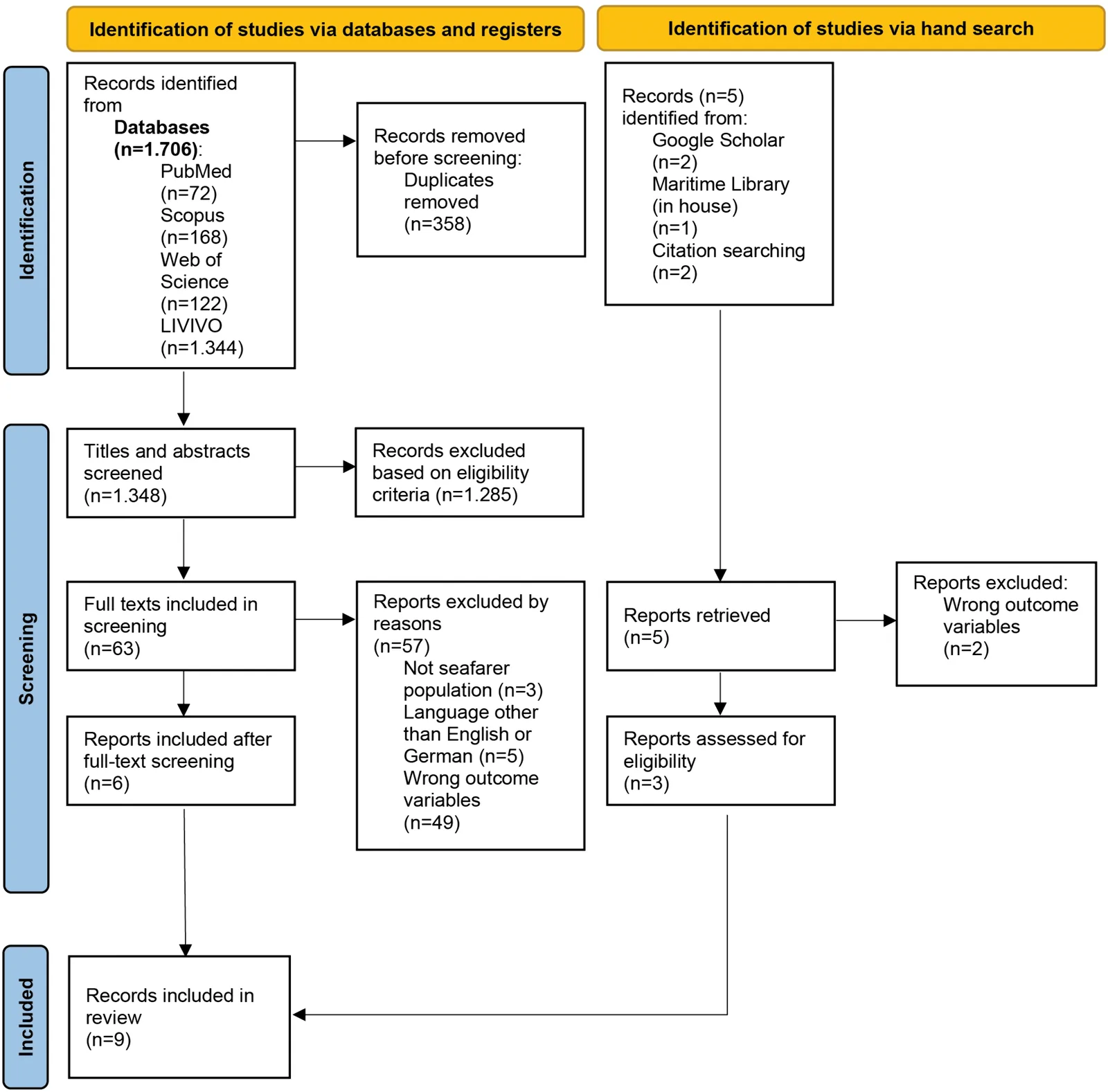
Prisma Flow Chart created according to Page et al. (2021) [27].

A total of 1.706 records were initially identified from electronic databases, and 5 records were found by hand search in Google Scholar, the institutional maritime library and citation searching. Duplicates were removed and subsequent screening applied through title and abstract review and full-text eligibility assessment. The PRISMA flow diagram in Fig 1 below illustrates the detailed process through these phases, resulting in the inclusion of 9 studies for qualitative analysis.

Eight of the nine included records are peer-reviewed articles, published in scientific journals and one is a master’s thesis, published online. The included articles refer to studies conducted in seven different countries: China (n = 1), Germany (n = 3), Iran (n = 1), Italy (n = 1), Republic of Korea (n = 1), Sweden (n = 1) and Philippines (n = 1). The publication dates range from 2019 to 2025 (Table 1).

**Table 1:** Overview of included literature.

*Primary target parameter*	*Titel*	*Authors*	*Publica-tion year*	Journal (Impact-Factor)	*Origin of affiliation*	*Study type*	*Type of approach*	*Quality (%)*
Mental health	Empowering seafarers as agents of their mental health: the role of information and communication technology in seafarers’ well-being	Abila et al. [19]	2023	Inquiry (2.3)	Philippines	Mixed methods: qualitative in-depth interviews and quantitative survey	Blended	100^1^
App-based health interventions	Possibilities, usage and needs of an app-based health prevention among seafarers	Arslan et al. [14]	2024	PLOS One (2.6)	Germany	Quantitative online survey	Blended	100^1^
Physical fitness	Development of physical training smartphone application to maintain fitness levels in seafarers	Battineni et al. [28]	2019	Int. Marit Health (1.0)	Italy	Interventional pilot study	Behavioral	20^1^
Health risks and proactive intervention	Human digital healthcare engineering for enhancing the health and well-being of seafarers and offshore workers: a comprehensive review	Cui et al. [31]	2025	MDPI Systems (3.1)	China	Comprehensive review	Structural	100^2^
Acceptance of digital health solutions	Smooth sailing: An exploratory study navigating the acceptance of digital health solutions and the impact on seafarer well-being	Herkommer & Siljevik Laine [30]	2023	/	Sweden	Qualitative interview study	Blended	100^2^
Skin cancer preventive behavior	Can adopting skin cancer preventive behaviors among seafarers be increased via a theory-based mobile phone-based text message intervention? A randomized clinical trial	Heydari et al. [18]	2021	BMC Public Health (3.6)	Iran	Randomized controlled trial (interventional)	Behavioral	100^1^
Physical activity	Feasibility of wearable digital healthcare devices among Korean male seafarers: a pilot study	Kim et al. [29]	2025	MDPI Healthcare (2.7)	Republic of Korea	Interventional pilot-study	Behavioral	0^1^
Nutrition	Seafarers’ attitudes and chances to improve the nutrition on merchant ships from the crews’ and cooks’ perspective	Neumann et al. [20]	2024	JOMT (2.7)	Germany	Quantitative survey study	Structural	80^1^
Need for digital health promotion	Pilot study on the development of digitally supported health promotion for seafarers on sea	Scheit et al. [17]	2025	Int. Marit Health (1.0)	Germany	Quantitative pilot study (survey and examinations)	Behavioral	60^1^

^1^Quality assessment using MMAT.

^2^Quality assessment using AACODS checklist.

The study groups in the included studies consist exclusively of seafarers on cargo vessels. No studies were found that included seafarers and other crew members on passenger ships. The concept of digital health literacy received no consideration in the reviewed evidence. Consequently, the inclusion of articles was limited to those addressing seafarers on cargo ships and the topics of digital health promotion and digital prevention.

The primary target parameters of the included sources encompass a range of digital health aims, such as interventions designed to modify specific health behaviors, including the enhancement of skin cancer preventive behavior [18] and the promotion of sufficient physical activity [28,29], as well as needs assessments on topics like nutrition [20] and digital applications [14,17,30], papers that call for the improvement of digital health promotion and prevention of mental health problems [19], and the presentation of innovative digital health options [31] (Table 1).

The evidence found can be roughly divided into methodological categories of interventional, survey and other studies (Tables 2–4). Needs analyses, multimodal assessments, feasibility and pilot testings, one randomized controlled trial (RCT) and one review were conducted.

**Table 2:** Population, concept and context (PCC) of interventional studies.

	*Population*			*Concept*				*Context*
*Authors*	*Description*	*Inclusion criteria*	*Exclusion criteria*	*Intervention*	*Media & Technology used*	*Outcome variables*	*Results*	*Description*
Battineni et al. [28]	28 participants in two pilot tests (1.: 13 land-based persons; 2.: 15 random seafarers); no further demographical information	Not mentioned	Not mentioned	Individual physical education based on BMI values and personal fitness levels	Mobile app (static app, that works offline)	Usability, feasibility, satisfaction, acceptability	Satisfaction and usability raised from first to second trial	2 weeks app usage per trial
Heydari et al. [18]	136 seafarers; IG^1^(n = 68) & CG^2^(n = 68); age range: ≤ 30–60 years), 100% male	Fluent in Persian, being seafarer (≥ 1 year), reside in trial area, have daily cellphone access	History of skin cancer	45 text messages in Persian were sent to IG daily; provided practical and simple health suggestions	Short message service (SMS)	Primary: change of skin cancer preventive behaviors; secondary: change of the PMT variable score	Intervention was effective for increasing skin cancer preventive behaviors; key PMT variables significantly increased: perceived self-efficacy, protection motivation, fear; significantly decreased: response costs, perceived rewards	45 days intervention with 1-month follow-up, study language: Persian, baseline data collection in maritime health center of Genaveh Port (Iran)
Kim et al. [29]	11 Korean male seafarers, average age: 47 ± 21.1 years; average BMI 24.8 ± 4.0 kg/m2	≥19 years; own smartphone, app literacy	Low app literacy, skin-related difficulties to wear the device	Tracking steps and calories burned via wearable; participants received feedback based on monitored activity	B.BAND (wrist-worn smartband), smartphone app, that is linked to internet platform	Physical activity (step counts, calorie consumption) adherence (daily wearing hours), device/app satisfaction, webpage access activation and failure	SBP/DBP^3^ decreased significantly; modest weight reduction (1.2 kg/12 weeks); moderate physical activity (5,729 steps/day); moderate device satisfaction; suboptimal adherence (14.2% of time not worn) with occasional data transmission problems	12-week period, monitoring took place onboard merchant ships

^1^Intervention group.

^2^Control group.

^3^Systolic and diastolic blood pressure.

**Table 3:** Population, concept and context (PCC) of survey studies.

	*Population*	*Concept*				*Context*
*Authors*	*Description*	*Description/ Methods*	*Media & Technology regarded*	*Outcome variables*	*Results*	*Description*
Arslan et al. [14]	976 seafarers, 99.4% male, age range: ≥ 19 - ≤ 60 years, 66.1% Filipinos	Online survey on technical requirements and user behavior for app-based health prevention, assuming digital tools can help overcome maritime-specific barriers to prevention	Health apps	Health app acceptance; areas of interest; cessation of app using; usage frequency/duration; well-being	52.5% seafarers had downloaded ≥1 health app, officers > ratings; interest: activity tracking (74.8%)> weight loss (41.8%)> getting taught exercises (41.0%); 51.1% discontinued – loss of interest (41.9%); usage: at home> at sea> in port; median WHO-5 score: 20.0	Onboard 65 merchant ships of one shipping company, study language: English
Neumann et al. [20]	970 respondents (96.5%), 810 crew members and 62 cooks included, mean age: crew 39.4 (SD 11.7) years, cooks 44.2 (SD 7.6) years; 100% male	Two paper-based questionnaires (cook and crew), assessed acceptance of digital health promotion, focused on supporting ship cooks to improve nutritional quality; using a tablet-based platform and nudging procedures	Digital tablet-based platform for ship cooks	Crew: well-being, nutrition and willingness to change habits; cooks: acceptance and willingness regarding digital tools; status of ship cooks’ training, daily routine of cooking	Crew: 98.8% value healthy eating for well-being, 83.7% Europeans and 90.7% Southeast Asians are willing to adapt eating habits. cook: are willing to use tablets (93.5%), to follow digital instructions despite extra work (87.1%); high interest in nutrition health (96.8%), nudging (95.2%), health feedback food (92.0%)	Onboard 68 merchant ships belonging to two German shipping companies, study language: English
Scheit et al. [17]	73 seafarers, mean age: ratings 40.4 (SD 11.8) years, officers 38.6 (SD 8.0) years, 100% male	Tablet-based digital survey, analysis of needs for digital health promotion	Health platform for ship crews	Addictive behavior, mental health, well-being, fatigue, health knowledge	31.9% were smokers; 80.6% consumed alcohol; 85.4% had moderate to severe depressive symptoms; well-being score 69.7%; fatigue elevated in 33.8% and daytime sleepiness (according to pupillometry) in 47.7%; health knowledge: < 60% correct answers (59.0% correct on sport, 50.2% on nutrition, 49.7% on fatigue)	Onboard three merchant vessels in presence of four scientific medical persons during three research voyages, study language: English

**Table 4:** Population, concept and context (PCC) of remaining studies.

	*Population*	*Concept*					*Context*
*Authors*	*Description*	*Description/ methods*	*Media & Technology regarded*	*Outcome variables*	*Recommended measure(s)*	*Results/ key insights*	*Description*
Abila et al.	**Interviews**: 26 individuals (seafarers, NGOs, maritime players, families), no mean age stated, 80.8% male **Survey**: 1,412 seafarers, mean age: 31, 96.7% male.	Mixed methods (two-phase strategy): qualitative interviews followed by quantitative survey; examination of seafarers’ MH and empowerment through ICT	ICT (including internet, smartphones, computers, web-based applications, MH apps, MH helplines and websites	Usage, acceptance, perceived usefulness/effectiveness Internet access	Providing reliable, easy-to-use, and cheap ICT infrastructure; suggesting formal education and training on MH, tele-counselling services, MH apps, MH helplines	Family-focused support is crucial: timely crew changes (80.8%), immediate family support (68.6%), communicating with families (87.8%); interventions related to own well-being were viewed as less useful, low use of digital MH tools	Onboard international merchant vessels, accessed remotely
Cui et al.	n.a.	Comprehensive review of the scope and potential of the Human Digital Healthcare Engineering (HDHE) framework to monitor health	Wearable sensors (health parameters), wireless transmission technologies, AI, specifically ML and DL	Description of HDHE framework	HDHE framework uses Human Digital Twin (HDT) for: 1) real-time health monitoring; 2) data transmission; 3) HDT-based analytics/simulations; 4) AI diagnosis; 5) predictive health analysis	HDHE: proactive solution for seafarers’ safety, health, well-being via HDT for real-time monitoring, predictive diagnostics, personalized care; challenges: technical/organizational complexities, privacy/security ethics, high costs.	Could affect seafarers globally
Herkom-mer & Siljevik Laine	18 participants, mean age and gender distribution not stated	Semi-structured qualitative interviews – grounded theory, Technology Acceptance Model (TAM) and Gioia’s analysis method	mHealth, applications, wearables, smartwatches, fitness trackers; Interviews via Google Meet	Acceptance of DHS: influences by perceived usefulness, ease of use, attitudes, well-being, external variables, and barriers	Organizations should design effective DHS and well-being strategies that comprehensively address all aspects of well-being	Three levels of DHS-acceptance (low, mid, high); crucial for well-being improvement (physical mental, social – holistic); DHS impacts well-being positively	Online videocall interviews

Table 2 summarizes the three interventional studies which examined health behavior and developed digital preventive or health promotional programs [18,28,29]. The sample size varied from 11 participants in a small-scale pilot study testing feasibility of wearable digital healthcare devices [29] to 136 participants in an RCT using 45 SMS text messages to increase skin cancer preventive behavior [18]. Whereas Battineni et al. conducted two pilot tests with a total of 28 participants to research the satisfaction and usability of a smartphone application for physical training [28]. Positive results can be observed in all three interventional studies. While the satisfaction and usability of Battineni et al.’s [28] smartphone app increased from first to second trial, the systolic (SBP) and diastolic blood pressure (DBP) in Kim et al.’s [29] wearable device trial decreased significantly and weight decreased modestly in the study sample during the 12-week intervention period. Participation adherence therefore was suboptimal in the examination of Kim et al., as the wrist-worn smart band was not worn 14.2% of the time, even though the instructions were to wear the device as consistently as possible. Heydari et al.’s RCT shows positive effects on skin cancer preventive behavior in the tested population and increased values in the variables of the Protection Motivation Theory (PMT), which means participants perceive skin cancer as severe, view themselves as being at personal risk, believe protective actions are effective, and believe they are capable of performing them after the intervention [18].

Three further articles employed questionnaire-based surveys to ascertain the needs and possibilities of digital health promotion onboard merchant ships [14,17,20] (Table 3). Sample size ranged from 73 seafarers in Scheit et al.’s pilot study [17] to 976 participants in Arslan et al.’s online survey [14]. The surveys were conducted both onboard cargo ships [17] as well as remotely operated, whether using paper-based questionnaires [20] or an online survey [14]. While Scheit et al. [17] examined seafarers’ needs for digital health promotion regarding digital health platforms for crews on board, Arslan et al. [14] analyzed possibilities, usage and needs for app-based prevention and Neumann et al. [20] assessed acceptance of digital health promotion with focus on ship cooks using a tablet-based platform, that supports cooking healthier and facilitates routines and procedures. The results of all the survey studies have shown that seafarers have a need for health promotion and prevention and that the interest in digital tools is high. Of all respondents, 52.5% already downloaded a health app before and 74.8% are interested in activity tracking [14]. Additionally, Scheit et al. showed that health knowledge was low. Less than 60% of knowledge questions were answered correctly [17]. Seafarers value healthy nutrition and are willing to adapt to their eating habits. Moreover, 93.5% of ship cooks would use a tablet-based platform and would follow instructions to cook healthier [20]. All included three survey studies were conducted from researchers of the same German institution.

The remaining three studies are summarized in Table 4. It contains a comprehensive review on the topic of human digital healthcare engineering (HDHE) [31], a mixed methods study that examined mental health empowerment [19], and a qualitative study that focused on the acceptance of digital health solutions [30]. Abila et al. conducted interviews with 26 participants and a survey with 1,412 seafarers. The mixed methods study examined seafarers’ mental health and empowerment through the usage of ICT. They found out that family-focused support is desired, whereas interventions related to their own well-being were rated as less useful and digital tools were seldomly used [19]. Herkommer and Siljevik Laine carried out semi-structured interviews regarding the acceptance of digital health solutions (DHS) and found out that DHS have positive impacts on well-being and therefore suggested organizations to design DHS and well-being strategies [30]. In Cui et al.’s comprehensive review the HDHE framework was presented, which used Human Digital Twins (DHT) to real-time monitor seafarers’ health in combination with simulations and predictive health analyses. The authors state that HDHE is a proactive solution for seafarers’ health and safety but that specific challenges concerning privacy and security as well as high costs exist [31].

### 3.2 Quality appraisal of included studies

Quality appraisal indicates substantial variability in methodological rigor across the included studies (Table 1). Among the three interventional studies [18,28,29] (Table 2), the two pilot trials achieved MMAT scores from 0 [29] to 20% [28], respectively, whereas the RCT [18] met all criteria with a MMAT score of 100%. The three survey studies [14,17,20] (Table 3) showed moderate to high quality: the two large-scale surveys with 970 and 976 respondents scored from 80 [20] to 100% [14], while the smaller pilot survey [17] with 73 participants achieved 60% in MMAT.

The remaining three studies [19,30,31] (Table 4) all demonstrate high methodological quality. Abila et al.’s [19] study fulfilled all 15 for mixed methods studies applied MMAT criteria, receiving a score of 100%. In addition, the comprehensive review from Cui et al. [31] and the qualitative masters’ thesis study from Herkommer and Siljevik Laine [30] were appraised using the AACODS checklist, with both studies achieving 100%, indicating strong credibility, relevance, and methodological transparency. A complete quality assessment using the MMAT and AACODS checklist can be found in the appendix (S2 and S3 Files).

### 3.3 Causes for conduct of studies

The authors of the included articles provide various reasons for conducting studies on health promotion and prevention among seafarers. Within this group, the prevalence of mental health issues, such as depressive symptoms, is high. Abila et al. report of elevated anxiety, stress and psychophysical exhaustion [19]. While Scheit et al. state the prevalence of moderate to severe depressive symptoms is 85.4%, the daytime sleepiness of seafarers is significantly increased (33.8%), compared to truck drivers [17]. The underlying references frequently cite fatigue and sleep problems as one of the main issues of maritime occupations [14,17,19,30,31]. Furthermore, cardiovascular diseases associated with increased metabolic risks are prevalent among seafarers. Thus, high rates of systolic and diastolic blood pressure, as well as significant overweight and obesity, can be observed [29]. As Heydari et al. emphasized, skin cancer and sunburn are conventional occupational diseases due to seafarers’ exposure to high levels of solar radiation. This is particularly relevant for seafarers who are required to work outside, such as deck ratings, especially in tropical or subtropical regions. [18]. Furthermore, seafarers frequently exhibit signs of high nicotine and alcohol consumption. Scheit et al. found that 31.9% of the participants were smokers, while 80.6% consumed alcohol in various types and dosages [17]. Neumann et al. draw attention to the issue of nutritional deficiencies among seafarers on merchant ships. It has been demonstrated that seafarers’ diets on board vessels are characterized by elevated levels of fat, sugar and calories. In comparison with their diets while on land, these diets exhibit a marked deficiency in the consumption of fresh fruits and vegetables [20]. Furthermore, Scheit et al. observe a deficiency in health knowledge among seafarers. The study examined health knowledge in relation to sports, nutrition and fatigue. The participants in the study only provided the correct answers in 59% of cases [17].

### 3.4 Covered media and technologies

Seven of the studies address the technologies of mHealth, including applications for smartphones and tablets. As referenced by Arslan et al. [14], the features encompassed a health app enabling activity tracking, weight loss, exercise and sleep tracking (Table 3). Meanwhile, Battineni et al. [28] utilized a BMI calculator and a fitness program in their mobile app (Table 2). Scheit et al. [17] address e-health applications and suggest the use of VR glasses and gamification to support digitally led sport competitions. Neumann et al. [20] concentrate on an online platform for food ordering and meal planning that is tablet-based and designed for use by ship’s cooks (Table 3). Furthermore, Abila et al. [19] propose the utilization of medical consultation services via helpline, in parallel with internet-based communication tools such as WhatsApp, email, and other messengers (Table 4). In the study from Kim et al. [29] a smartphone application to connect wearable devices with the smartphones of seafarers was employed (Table 2). Herkommer and Siljevik Laine [30] explored smartphone applications, wearables, online video consultation platforms, and tools incorporating gamification and reward systems (Table 4).

### 3.5 Behavioral vs. structural approaches

The identified articles can be distinguished regarding their focus on training and knowledge acquisition, as well as environmental and organizational concepts or ideas. These articles employ behavioral (n = 4) or structural approaches (n = 2), respectively, with three studies adopt blended strategies that address both individual and organizational levels (Table 1).

#### Behavioral approaches.

Four of the included studies [17,18,28,29] focus on interventions aimed at changing individual behaviors and enhancing personal responsibility for health. For example, Battineni et al. developed a physical education program tailored to seafarers’ BMI, encouraging personalized activity plans. Heydari et al. implemented a theory-based SMS intervention, sending daily messages to increase self-efficacy and motivation for skin cancer prevention among seafarers. Kim et al. explored the feasibility of self-monitoring using wearable devices, enabling seafarers to track their steps and calories burned, with feedback delivered via a mobile application (Table 2). Scheit et al. assessed individual coping strategies for stress management and recommended future interventions to prioritize the prevention of addictive behavior, promotion of sports, sleep hygiene, and mental health. The authors suggested the usage of digital tools included health apps, VR glasses, gamification, and digitally supported sporting competitions.

#### Structural approaches.

Two articles [20,31] emphasize environmental and organizational changes to support health. Cui et al. introduced the Human Digital Healthcare Engineering (HDHE) framework, utilizing Human Digital Twin technology to remotely monitor seafarers’ health and deliver targeted interventions. While Neumann et al. demonstrated that seafarers’ dietary behaviors could be improved through structural modifications, such as equipping ship cooks with a tablet-based digital platform to facilitate healthy cooking and health promotion, as well as implementing nudging strategies and modified food ordering processes.

#### Blended approaches.

In three articles [14,19,30] both behavioral and structural elements were integrated. Abila et al. highlighted the importance of providing reliable, user-friendly, and affordable ICT infrastructure, alongside formal education, tele-counseling, mental health apps, and helplines. Their approach emphasizes empowering seafarers as active agents in managing their mental health, rather than passive recipients of care. Arslan et al. identified maritime-specific barriers, such as limited internet connectivity and recreational time, as significant obstacles to sustained health app use. The authors suggest that shipping companies can play a key role by providing internet access, health education, guidance on app selection, and licenses for paid apps, so that seafarers are empowered to take responsibility for their own health. The Technology Acceptance Model (TAM) was applied by Herkommer and Siljevik Laine to examine factors influencing the use of digital health tools, underscoring the need for organizations to design user-friendly technologies and comprehensive well-being strategies that address all aspects of seafarers’ health.

### 3.6 Digital health promotion programs for seafarers

As scientific evidence in the field of digital health promotion and prevention measures for seafarers is modest, this scoping review is supplemented with examples of digital health intervention programs that are available online but have not been scientifically assessed or published in peer-reviewed literature.

Six digital health programs specifically designed for seafarers and crew members on merchant ships were identified. These programs are provided by international welfare organizations, shipping companies, and maritime health institutions or companies, and address a variety of health topics relevant to life at sea, including physical activity and training, mental health, and overall health promotion. Table 5 summarizes key characteristics of selected digital health promotion programs, including their primary focus, responsible organizations, health topics covered, accessibility, and target groups.

**Table 5:** Digital health promotion programs for seafarers.

*Primary target parameter*	*Program title*	*Responsible organization*	*Health topics*	*Access-ibility*	*Target group*	*Source*
Help and support, health and wellbeing	**ISWAN for Seafarers**	The International Seafarers’ Welfare and Assistance Network	Mental health, physical activity, dental care, nutrition, relaxation, participation, illness prevention, stress, sleep, hydration	Free	Seafarers, yacht crew members and their families	[15]
Sport compete-tions	**FIT4SEA**	Sea Health & Welfare	Annual sports competition (disciplines: running, walking, cycling, cross-training, rowing, weight training)	Restricted	All seafarers on Danish flagged vessels	[32]
Mental health and wellbeing	**(My) Wellness at Sea**	Sailors’ Society	Five key areas on individual wellbeing: social, emotional, physical, intellectual, spiritual wellness	Free after registra-tion	All seafarers	[16]
Health behavior	**WellAtSea**	WellAtSea Limited	Wellness, fitness, participation, knowledge, mental health	Restricted	Seafarers from shipping companies that order the program	[33]
Several health-related subcategories	**Crew-Healthy**	Institute for Occupational and Maritime Medicine (ZfAM) of the University Medical Hospital Hamburg-Eppendorf (UKE)	seven educational sections: sport & physical health, stress & mental health, occupational health & ergonomics, sleep & fatigue, general health and COVID-19	Restricted	Seafarers	[34]
Happiness	**Happy at Sea**	The Mission to Seafarers	Happiness, welfare, mental well-being	Free	All seafarers	[35]

These programs cover a broad spectrum of health topics, including mental health and happiness, physical activity, nutrition, stress management, sleep, and general wellbeing. Accessibility varies, with some programs were freely available to all seafarers and others restricted to specific companies or nationality of the vessels. The target groups are similarly diverse, ranging from all seafarers, other crew members onboard and their families to those employed by particular shipping companies.

All identified programs are designed to be accessible through digital platforms, with varying modes of delivery. ISWAN for Seafarers provides health resources via both a mobile app and a web platform [15]. FIT4SEA is accessible through its dedicated website [32], while (My) Wellness at Sea offers both a website and a mobile app for users [16]. WellAtSea is delivered as an online tool, though it is not specified whether this is web- or app-based [33]. Happy at Sea is accessible via a mobile app [35]. CrewHealthy is unique because it operates as a browser-based e-health platform that can function without an active internet connection, ensuring usability even in low-connectivity environments [34].

Except for CrewHealthy, all programs are accessible without physical setting restrictions, allowing seafarers and crew members of merchant ships to use these resources both at sea and while ashore. In contrast, CrewHealthy is only available onboard participating vessels, limiting its use to those specific shipboard environments. Furthermore, all programs are provided in English, ensuring broad accessibility for the international maritime workforce.

## 4. Discussion

### 4.1 Study characteristics

This scoping review provides a comprehensive overview of the current landscape of digital health promotion and prevention measures for seafarers’ onboard merchant ships, combining evidence from peer-reviewed literature and grey literature, as well as online digital health programs. Even though the aim of the review was to map evidence about digital health promotion, prevention and health literacy in the population of seafarers and other crew members on board merchant ships, the results show a significant gap within this field. No studies were found that included crews on passenger ships. Moreover, the concept of digital health literacy received no consideration in the reviewed evidence. Consequently, this scoping review is unable to furnish a more comprehensive overview of articles that address those topics and thus was limited to those addressing seafarers on cargo ships and the topics of digital health promotion and digital prevention.

The systematic search and selection process resulted in the inclusion of nine studies, reflecting the emerging but still limited evidence base in this field. While there is a somewhat broader literature base on health promotion and prevention for seafarers in general [36], studies specifically addressing digital approaches remain scarce. Most of the existing research is foundational [14,17].

The examined sources were published between 2019 and 2025, with seven of nine appearing in the last three years (2025 (n = 3), 2024 (n = 2) and 2023 (n = 2)) as shown in Table 1, indicating a recent increase in interest and activity in this area.

The included studies represent a diverse international perspective, with research conducted in China, Germany, Iran, Italy, Republic of Korea, Sweden, and the Philippines. This geographic spread highlights the global relevance of digital health for the maritime sector.

In terms of content and primary target parameters as well as of methodology, the evidence base is heterogeneous. Thematically, the studies ranged from interventions targeting skin cancer preventive behavior [18] to physical activity [28,29], over broader needs assessments concerning nutrition [20] or digital health promotion [17] or applications [14], to theoretical frameworks to address early health promotion [31], and studies calling for improved digital mental health promotion [19,30]. Generally, seven of the included studies focused on mHealth, utilizing smartphones and tablets, indicating that mobile programs are considered the most promising approaches. Methodologically, the included studies comprise interventional designs, survey-based needs assessments, a comprehensive review, a mixed methods study, and a qualitative investigation. This diversity reflects both the early stage of research in this field and the exploratory nature of many current projects. This is consistent with previous observations that most research on digital health for seafarers is still foundational, with few robust evaluations of effectiveness or long-term impact [7,19].

The review also identified six practical digital health programs, which are specifically designed for seafarers. These programs, often developed by maritime organizations or commercial companies, address a wide range of health topics. While these offerings demonstrate practical needs, innovation and a growing commitment to supporting seafarers’ health, their effectiveness and user acceptance remain largely unstudied [7]. This highlights the need for future studies to move beyond needs assessments and pilot testing, and to conduct rigorous evaluations of digital health interventions in real-world maritime settings.

Furthermore, the review underscores the importance of considering both behavioral and structural factors in the design and implementation of digital health solutions. Barriers such as limited connectivity, harsh environmental conditions, hierarchical culture, and data privacy concerns must be addressed to ensure successful adoption and sustained use of digital health tools at sea.

### 4.2 Quality assessment

The quality assessment via MMAT and AACODS revealed substantial differences in the methodological rigor of the included studies. While five studies obtained a quality score of 100% [14,18,19,30,31], one study received a rating of 0% [29], another was rated with 20% [28], and the remaining two studies received ratings of 60% and 80% [17,20], respectively. This results from the fact that most included studies are pilot or feasibility studies with limited generalizability. Nonetheless, they are valuable given the scarcity of research and the hard-to-reach nature of the seafaring population. Intervention studies in this context are difficult to conduct and often face high data loss. Standard quality appraisal tools like the MMAT are not ideally suited for such heterogeneous and early-stage research, especially when items from the MMAT are not reported in the articles, making their results less meaningful [37]. Nevertheless, these studies provide important new insights and lay the groundwork for future research. These results further underscore the need for more standardized and methodologically robust research in the field of digital health promotion and prevention for seafarers.

### 4.3 Potentials of digital health tools

The articles reviewed identify several key potentials of digital health tools for seafarers on merchant ships. These technologies enable a shift from reactive to proactive health management. To that aim, Cui et al. promoted utilizing predictive diagnostics, such as digital healthcare engineering and deploying digital twins [31], whereas Kim et al. applied wearable devices and a self-monitoring system [29]. The objective of both concepts was facilitating early intervention and health promotion. As Abila et al. indicated, a notable benefit of mobile technology is its potential to enhance seafarers’ empowerment. Mobile apps and monitoring systems provide seafarers with personalized feedback, knowledge, and tools for autonomous health management, thereby increasing their self-awareness [19].

Digital platforms also facilitate targeted education and deliver context-specific content, for example platforms designed for ship cooks to promote healthy nutrition onboard, like Neumann et al. promoted [20], or tailored fitness programs, like the app “Wellness on Ship” (WOS), developed by Battineni et al. [28]. Additionally, features like gamification and reward systems can foster a sense of belonging and unity among crew members, encouraging social interaction [17,30].

Ensuring reliable connectivity at sea is instrumental in facilitating direct and continuous access to digital health solutions [14]. From an organizational perspective, these tools can reduce human error and accidents, while also helping to attract and retain skilled workers by creating a safer and more supportive work environment [17,30,31].

### 4.4 Maritime-specific barriers of digital health solutions

The implementation and utilization of digital health technologies onboard merchant ships face several maritime-specific barriers. The unique physical environment presents significant challenges, including frequent connectivity limitations. Many seafarers experience unreliable, slow, or expensive internet access, with around 15% reporting no access at all [14,19]. Digital hardware must also withstand harsh maritime conditions such as humidity, saltwater corrosion, ship movements, mechanical vibration, and temperature fluctuations [31]. Additionally, vessel-specific safety standards require digital equipment to meet stringent regulations, especially on ships like oil tankers where it is essential to mitigate any potential risk of fire, making these tools more expensive and difficult to implement [30].

Occupational and organizational culture further complicate adoption. A prevailing “ship first” mentality prioritizes operational safety and efficiency over individual health needs [19]. Cultural factors, such as a “macho culture” and taboos around discussing mental health, discourage openness and help-seeking [19,30]. Seafarers may hesitate to adopt digital tools due to concerns that their personal data could be used by superiors for surveillance or control purposes [30].

Structural and logistical constraints include high workloads and lack of time, which are primary reasons seafarers discontinue using health apps [14]. The multinational nature of crews, with diverse languages, cultures, and dietary preferences, means that standardized solutions often fail to meet everyone’s needs [20].

Finally, data privacy and legal issues are decisive. Seafarers may fear constant monitoring and loss of confidentiality, leading to altered behavior because of feeling observed (the “Hawthorne Effect”) and retention to use digital health tools [30,31]. Sharing personal health data across international jurisdictions also raises complex legal challenges [31].

### Strengths and limitations

A key strength of this article is its comprehensive and systematic approach, which included a scoping review including both peer-reviewed and grey literature, as well as a demonstration of digital health programs from practice available online. By applying broad inclusion criteria and searching multiple databases and sources, the review provides a thorough overview of the current landscape of digital health promotion and prevention for seafarers. The use of the JBI methodology in accordance with the PRISMA guidelines and the prospective protocol registration in OSF further enhance the transparency and reproducibility of the review process.

However, limitations that should be acknowledged exist. First, the present scoping review was conducted by a single reviewer, which may have increased the risk of selection bias and data extraction errors. Despite the implementation of predefined eligibility criteria, standardized extraction procedures, and consultation on uncertain cases to enhance consistency, the absence of independent second screening constitutes a methodological limitation. Second, the body of research on this topic is very limited and the evidence base identified was highly heterogeneous, with most included studies being small-scale, pilot, or feasibility studies, and only one randomized controlled trial. This limits the generalizability of the findings and precludes robust conclusions about the effectiveness of specific interventions. Third, the use of standard quality appraisal tools, such as the MMAT and AACODS checklist, is challenging in such a diverse evidence landscape and may not fully capture the value of early-stage or descriptive research. Fourth, the body of the evidence included was limited to publications published in English and German, which may have excluded studies from major seafaring nations. Finally, the rapidly evolving nature of digital health means that some recent or unpublished initiatives may not have been captured.

Furthermore, it should be recognized that three of the included studies were conducted by members of the German Institute for Occupational and Maritime Medicine of the University Medical Center Hamburg-Eppendorf, where the authors and co-authors are employed.

Despite these limitations, this review offers important new insights into the opportunities and challenges of digital health interventions for seafarers and highlights critical gaps for future research and practice in this underexplored field.

### Conclusion

In summary, the evidence base for digital health promotion and prevention among seafarers is still modest and methodologically as well as thematically diverse. No evidence was found on the target group of crews on passenger ships, as well as on the topic of digital health literacy on merchant ships. Nevertheless, the frequency of publications in this field appears to be increasing in recent years. Therefore, this review appears to indicate two things. First, there is a growing interest in the field and second, there is an urgent need for more robust research. The inclusion of non-scientifically assessed digital health programs provides a more complete picture of the current landscape and highlights the proactive efforts of stakeholders to address the unique health challenges faced by seafarers. Consequently, future research should prioritize the evaluation of existing digital interventions, address implementation barriers, and explore strategies to maximize the reach and effectiveness of digital health solutions in the maritime sector.

## Supporting information

S1 File Search strategy. (DOCX)

S2 File Quality assessment MMAT table. (DOCX)

S3 File Quality assessment AACODS table. (DOCX)

S4 File PRISMA-ScR-Checklist. (DOCX)

## References

[1] Lefkowitz RY, Slade MD. Seafarer mental health study. London: ITF Seafarers’ Trust; 2019.

[2] Sampson H, Ellis N, Acejo I, Turgo N, Tang L. The working and living conditions of seafarers on cargo ships in the period 2011-2016. Cardiff: Seafarers International Research Centre (SIRC) I1 - Cardiff University; 2018. Report No.: 1-900174-50-2.

[3] Graham CAE. Maritime security and seafarers’ welfare: towards harmonization. WMU J Marit Aff. 2009;8(1):71–87. doi: 10.1007/bf03195154

[4] Carrera-Arce M, Baumler R, Bhatia BS, Du L. Shore leave: rare, brief and in danger of extinction. Malmö: World Maritime University; 2025.

[5] Sampson H, Ellis N. Seafarer’s mental health and wellbeing. Institutions of Occupational Safety and Health; 2019.

[6] Oldenburg M, Jensen H-J, Latza U, Baur X. Seafaring stressors aboard merchant and passenger ships. Int J Public Health. 2009;54(2):96–105. doi: 10.1007/s00038-009-7067-z 19288290

[7] Arslan LC, Dengler D, Belz L, Neumann FA, Zyriax B-C, Harth V, et al. Exploration of seafarers’ mobile proficiency as a prerequisite for possible health app-based health promotion on board. Inquiry. 2023;60:469580231206264. doi: 10.1177/00469580231206264 37909669 PMC10621288

[8] Mohammadzadeh N, Gholamzadeh M. Requirements, challenges, and key components to improve onboard medical care using maritime telemedicine: narrative review. Int J Telemed Appl. 2023;2023:9389286. doi: 10.1155/2023/9389286 37362154 PMC10287522

[9] World Health Organization. WHO guideline recommendations on digital interventions for health system strengthening: evidence and recommendations. Geneva: World Health Organization; 2019. 31162915

[10] World Health Organization. Global strategy on digital health 2020-2025. Geneva: World Health Organization; 2021.

[11] World Health Organization. Ottawa charter for health promotion. Geneva: World Health Organization; 1986.

[12] World Health Organization. Health promotion glossary. Geneva: World Health Organization; 1998.

[13] Sørensen K, Van den Broucke S, Fullam J, Doyle G, Pelikan J, Slonska Z, et al. Health literacy and public health: a systematic review and integration of definitions and models. BMC Public Health. 2012;12:80. doi: 10.1186/1471-2458-12-80 22276600 PMC3292515

[14] Arslan L, Dengler D, Belz L, Neumann FA, Zyriax B-C, Harth V, et al. Possibilities, usage and needs of an app-based health prevention among seafarers. PLoS One. 2024;19(11):e0310440. doi: 10.1371/journal.pone.0310440 39602460 PMC11602079

[15] International Seafarers Welfare Assistance Network (ISWAN). Our app: International Seafarers’ Welfare and Assistance Network; 2026. Available from: https://www.iswan.org.uk/our-app/

[16] Sailors Society. Wellness at Sea: MyWellnessAtSea.org. Available from: https://mywellnessatsea.org/index

[17] Scheit L, Dengler D, Belz L, Reck C, Harth V, Oldenburg M. Pilot study on the development of digitally supported health promotion for seafarers on sea. Int Marit Health. 2025;76(1):1–10. doi: 10.5603/imh.102259 39868605

[18] Heydari E, Dehdari T, Solhi M. Can adopting skin cancer preventive behaviors among seafarers be increased via a theory-based mobile phone-based text message intervention? A randomized clinical trial. BMC Public Health. 2021;21(1):134. doi: 10.1186/s12889-020-09893-x 33446158 PMC7807693

[19] Abila S, Kitada M, Malecosio S, Tang L, Subong-Espina R. Empowering Seafarers as agents of their mental health: the role of information and communication technology in seafarers’ well-being. Inquiry. 2023;60. 10.1177/00469580231162752 PMC10037725 36950721

[20] Neumann FA, Belz L, Dengler D, Harth V, Reck C, Oldenburg M, et al. Seafarers’ attitudes and chances to improve the nutrition on merchant ships from the crews’ and cooks’ perspective. J Occup Med Toxicol. 2024;19(1):13. doi: 10.1186/s12995-024-00412-x 38698394 PMC11067207

[21] Peters MDJ, Marnie C, Tricco AC, Pollock D, Munn Z, Alexander L, et al. Updated methodological guidance for the conduct of scoping reviews. JBI Evid Synth. 2020;18(10):2119–26. doi: 10.11124/JBIES-20-00167 33038124

[22] Tricco AC, Lillie E, Zarin W, O’Brien KK, Colquhoun H, Levac D, et al. PRISMA extension for scoping reviews (PRISMA-ScR): checklist and explanation. Ann Intern Med. 2018;169(7):467–73. doi: 10.7326/M18-0850 30178033

[23] Reck C, Belz L, Dengler D, Harth V, Puls N, Oldenburg M. Digital health promotion, prevention and health literacy measures among seafarers and crews on cargo and passenger ships: a scoping review protocol. 2025. 10.1371/journal.pone.0356798 PMC13513975 42647484

[24] Ouzzani M, Hammady H, Fedorowicz Z, Elmagarmid A. Rayyan-a web and mobile app for systematic reviews. Syst Rev. 2016;5(1):210. doi: 10.1186/s13643-016-0384-4 27919275 PMC5139140

[25] Hong QN, Fàbregues S, Bartlett G, Boardman F, Cargo M, Dagenais P, et al. The Mixed Methods Appraisal Tool (MMAT) version 2018 for information professionals and researchers. EFI. 2018;34(4):285–91. doi: 10.3233/efi-180221

[26] Tyndall J. AACODS Checklist for appraising grey literature. Flinders University; 2010.

[27] Page MJ, McKenzie JE, Bossuyt PM, Boutron I, Hoffmann TC, Mulrow CD, et al. The PRISMA 2020 statement: an updated guideline for reporting systematic reviews. BMJ. 2021;372:n71. doi: 10.1136/bmj.n71 33782057 PMC8005924

[28] Battineni G, Di Canio M, Chintalapudi N, Amenta F, Nittari G. Development of physical training smartphone application to maintain fitness levels in seafarers. Int Marit Health. 2019;70(3):180–6. doi: 10.5603/IMH.2019.0028 31617935

[29] Kim D-R, Park J-H, Jang M-W, Sung M-J, Song S-H, Huh U, et al. Feasibility of wearable digital healthcare devices among Korean male seafarers: a pilot study. Healthcare (Basel). 2025;13(10):1176. doi: 10.3390/healthcare13101176 40428012 PMC12111138

[30] Herkommer C, Siljevik Laine S. Smooth sailing: An exploratory study navigating the acceptance of digital health solutions and the impact on seafarer well-being [Student thesis]; 2023.

[31] Cui MX, He K, Wang F, Paik JK. Human digital healthcare engineering for enhancing the health and well-being of seafarers and offshore workers: a comprehensive review. Systems. 2025;13:335.

[32] Sea Health & Welfare. FIT4SEA. Available from: https://fit4sea.shw.dk/welcome

[33] WellAtSea Limited. WellAtSea - Full service implementation for seafarer well-being: WellAtSea.com. Available from: https://wellatsea.com/

[34] Institute for Occupational and Maritime Medicine UMHH-E. e-healthy ship: results: e-healthy ship EU Project. Available from: https://www.e-healthy-ship.eu/en/results/

[35] The Mission to Seafarers. Happy at Sea | The Seafarers App by The Mission to Seafarers. Available from: https://www.missiontoseafarers.org/our-app

[36] Carter T, Karlshoej K. The design of health promotion strategies for seafarers. Int Marit Health. 2017;68(2):102–7. doi: 10.5603/IMH.2017.0019 28660613

[37] Hong QN, Gonzalez-Reyes A, Pluye P. Improving the usefulness of a tool for appraising the quality of qualitative, quantitative and mixed methods studies, the Mixed Methods Appraisal Tool (MMAT). J Eval Clin Pract. 2018;24(3):459–67. doi: 10.1111/jep.12884 29464873

