# Supplementary material for: Digital health promotion, prevention and health literacy measures among seafarers and crews on cargo and passenger ships: A scoping review
Source: PLoS One. 2026 Aug 26;21(8):e0356798. doi: 10.1371/journal.pone.0356798 (PMC13513975; doi:10.1371/journal.pone.0356798)
Supplement: Supplementary file 1 [file pone.0356798.s001.docx]

## S1: Search strategy

### SCOPUS

TITLE-ABS-KEY((seafarer* OR sailor* OR "ship crew*" OR "ship personnel*" OR seaman OR seamen) AND (ship* OR "merchant vessel*" OR ferry OR ferries OR "cruise ship*" OR "at sea*" OR onboard OR "on board" OR "merchant marine*" OR seagoing) AND ("health literacy" OR "health competence" OR "health education" OR "health promotion" OR prevention OR “health measure*” OR “health intervention*” OR “health program” OR ehealth OR “electronic health” OR mhealth OR "mobile health" OR dhealth OR “digital health”))

AND (PUBYEAR > 2014 AND PUBYEAR < 2026)

AND (LANGUAGE(english) OR LANGUAGE(german))

Scopus data bases / “Subject areas”: Medicine, Social Sciences, Computer Science, Arts and Humanities, Psychology, Health Professions

Conducted on August, 14 2025

Number of records retrieved: 168

### PubMed

((seafarer*[tiab] OR sailor*[tiab] OR "ship crew*"[tiab] OR "ship personnel*"[tiab] OR seaman[tiab] OR seamen[tiab]) AND (ship*[tiab] OR "merchant vessel*"[tiab] OR ferry[tiab] OR ferries[tiab] OR "cruise ship*"[tiab] OR "at sea"[tiab] OR onboard[tiab] OR "on board"[tiab] OR "merchant marine*"[tiab] OR seagoing[tiab]) AND ("health literacy"[MeSH Terms] OR "health literacy"[tiab] OR "health competence"[tiab] OR "health education"[MeSH Terms] OR "health education"[tiab] OR "health promotion"[MeSH Terms] OR "health promotion"[tiab] OR prevention[tiab] OR "health measure*"[tiab] OR “health intervention*”[tiab] OR "health program"[tiab] OR ehealth[tiab] OR "electronic health"[tiab] OR mhealth[tiab] OR "mobile health"[tiab] OR dhealth[tiab] OR "digital health"[tiab])) AND ("2015/01/01"[Date - Publication] : "2025/12/31"[Date - Publication]) AND (english[lang] OR german[lang])

Conducted on August, 14 2025

Number of records retrieved: 72

### Web of Science

TS=((seafarer* OR sailor* OR "ship crew*" OR "ship personnel*" OR seaman OR seamen) AND (ship* OR "merchant vessel*" OR ferry OR ferries OR "cruise ship*" OR "at sea*" OR onboard OR "on board" OR "merchant marine*" OR seagoing) AND ("health literacy" OR "health competence" OR "health education" OR "health promotion" OR prevention OR "health measure*" OR “health intervention*” OR "health program" OR ehealth OR "electronic health" OR mhealth OR "mobile health" OR dhealth OR "digital health"))

Query link: https://www.webofscience.com/wos/woscc/summary/0fcfd2c0-e5b5-4d13-be13-890a40a830df-0173f3c39e/relevance/1

Filters:

Publication date: 01.01.2015 – 31.12.2025

Language: English, German

Conducted on August, 14 2025

Number of records retrieved: 122

##### LIVIVO

(seafarer* OR sailor* OR "ship crew*" OR "ship personnel*" OR seaman OR seamen) AND (ship* OR "merchant vessel*" OR ferry OR ferries OR "cruise ship*" OR "at sea" OR onboard OR "on board" OR "merchant marine*" OR seagoing) AND ("health literacy" OR "health competence" OR "health education" OR "health promotion" OR prevention OR "health measure*" OR “health intervention*” OR "health program" OR ehealth OR "electronic health" OR mhealth OR "mobile health" OR dhealth OR "digital health")

Filter:

Published: 2015-2025

Conducted on August, 14 2025

Number of records retrieved: 1.344
