# Supplementary material for: Digital health promotion, prevention and health literacy measures among seafarers and crews on cargo and passenger ships: A scoping review
Source: PLoS One. 2026 Aug 26;21(8):e0356798. doi: 10.1371/journal.pone.0356798 (PMC13513975; doi:10.1371/journal.pone.0356798)
Supplement: Supplementary file 2 [file pone.0356798.s002.docx]

## S2 Mixed Methods Appraisal Tool (MMAT)

|  | | | **SCREENING QUESTIONS** | | **1. Qualitative studies** | | | | | **Score (%)** |
| --- | --- | --- | --- | --- | --- | --- | --- | --- | --- | --- |
| RefID | First author | Year | S1. Are  there clear  research  questions? | S2. Do the  collected  data allow  to address  the  research  questions? | 1.1. Is the  qualitative  approach  appropriate to  answer the  research  question? | 1.2. Are the  qualitative  data collection  methods  adequate to  address the  research  question? | 1.3. Are the  findings  adequately  derived from  the data? | 1.4. Is the  interpretation of  results  sufficiently  substantiated by  data? | 1.5. Is there  coherence  between  qualitative data  sources,  collection,  analysis and  interpretation? |  |
| / |  |  |  |  |  |  |  |  |  |  |
|  | | | | | **2. Quantitative randomized controlled trials** | | | | |  |
|  |  |  |  |  | 2.1. Is  randomization  appropriately  performed? | 2.2. Are the  groups  comparable at  baseline? | 2.3. Are there  complete  outcome data? | 2.4. Are  outcome  assessors  blinded to the  intervention  provided? | 2.5 Did the  participants  adhere to the  assigned  intervention? |  |
| 18 | Heydari et al. | 2021 | Yes | Yes | Yes | Yes | Yes | Yes | Yes | 100 |
|  | | | | | **3. Quantitative non-randomized studies** | | | | |  |
|  |  |  |  |  | 3.1. Are the participants representative of the target population? | 3.2. Are measurements appropriate regarding both the outcome and intervention (or exposure)? | 3.3. Are there complete outcome data? | 3.4. Are the confounders accounted for in the design and analysis? | 3.5. During the study period, is the intervention administered (or exposure occurred) as intended? |  |
| 29 | Kim et al. | 2025 | Yes | Yes | No | Can’t tell | No | Can’t tell | Can’t tell | 0 |
|  | | | | | **4. Quantitative descriptive studies** | | | | |  |
|  |  |  |  |  | 4.1. Is the  sampling  strategy  relevant to  address the  research  question? | 4.2. Is the  sample  representative  of the target  population? | 4.3. Are the  measurements  appropriate? | 4.4. Is the risk  of nonresponse  bias low? | 4.5. Is the  statistical  analysis  appropriate to  answer the  research  question? |  |
| 14 | Arslan et al. | 2024 | Yes | Yes | Yes | Yes | Yes | Yes | Yes | 100 |
| 28 | Battineni et al. | 2019 | Yes | Yes | No | No | Can’t tell | Can’t tell | Yes | 20 |
| 20 | Neumann et al. | 2024 | Yes | Yes | Yes | Yes | Can’t tell | Yes | Yes | 80 |
| 17 | Scheit et al. | 2025 | Yes | Yes | Yes | Can’t tell | Yes | Can’t tell | Yes | 60 |
|  | | | | | **5. Mixed methods studies** | | | | |  |
|  |  |  |  |  | 5.1. Is there an  adequate  rationale for  using a mixed  methods design | 5.2. Are the  different  components of  the study  effectively | 5.3. Are the  outputs of the  integration of  qualitative and  quantitative | 5.4. Are  divergences and  inconsistencies  between  quantitative and | 5.5. Do the  different  components of  the study  adhere to the |  |
| 19 | Abila et al. | 2023 | Yes | Yes | Yes | Yes | Yes | Yes | Yes | 100 |

Reference:

Hong QN, Fàbregues S, Bartlett G, Boardman F, Cargo M, Dagenais P, et al. The mixed methods appraisal tool (MMAT) version 2018 for information professionals and researchers. Education for Information. 2018;34(4):285-91.Accessed via: http://mixedmethodsappraisaltoolpublic.pbworks.com/w/file/fetch/140056890/Reporting the results of the MMAT.pdf.
