# Supplementary material for: Digital health promotion, prevention and health literacy measures among seafarers and crews on cargo and passenger ships: A scoping review
Source: PLoS One. 2026 Aug 26;21(8):e0356798. doi: 10.1371/journal.pone.0356798 (PMC13513975; doi:10.1371/journal.pone.0356798)
Supplement: Supplementary file 3 [file pone.0356798.s003.docx]

## S3 AACODS Checklist (Authority, Accuracy, Coverage, Objectivity, Date, Significance)

| Reference | | | AACODS | | | | | | Score % |
| --- | --- | --- | --- | --- | --- | --- | --- | --- | --- |
| RefID | Authors | Year | Authority | Accuracy | Coverage | Objectivity | Date | Significance |  |
| 31 | Cui et al. | 2025 | Yes | Yes | Yes | Yes | Yes | Yes | 100 |
| 30 | Herkommer & Siljevik Laine | 2023 | Yes | Yes | Yes | Yes | Yes | Yes | 100 |

Reference:

Tyndall J. AACODS Checklist for appraising grey literature. Flinders University. 2010.
